# Account of a Case of Trismatic Tetanus, Produced by the Passage of Large, Rough, and Angular Pieces of Clay from the Intestinal Canal into the Vagina, and Cured by Tobacco Injections

**Published:** 1831-02

**Authors:** Burleigh Smart

**Affiliations:** Kennebunk, Maine.


### TRISMATIC TETANUS.

*Account of a Case of Trismatic Tetanus, produced by the Passage of large, rough, and angular Pieces of Clay from the Intestinal Canal into the Vagina, and cured by Tobacco Injections.* By BURLEIGH SMART, M.D. of Kennebunk, Maine. (Read before the Medical Society of Maine, at its semi-annual meeting at Portland, in January 1825.)*

IN March 1824, I was requested to visit R. S., an unmarried female, aged twenty-two years. On my entrance into her room, I was struck with the view of a pale, ghastly visage; the eyes rolled up, the angles of the mouth retracted, nostrils drawn upward, and the cheeks backward, mouth closed; exhibiting altogether an expression of indescribable suffering. She was lying extended on her back, her head and heels only touching the bed, the spine recurvated into an arch of almost a semicircle; her jaws were locked. In about a minute the spasm subsided, and she swallowed, with some difficulty, a teaspoonful of Oleum Terebinthinæ, from which she experienced immediate relief. The dose of oil was repeated in about five minutes, in consequence of a partial return of the *cramp*, (as the attendants termed it,) which was that violent constriction and pain, darting from the ensiform cartilage to the spine and extensor muscles of the neck.

* American Journal of Med. Sciences.

During the intervals of the cramp, as she had but a few slight returns of the trismatic and tetanic spasms, she gave me the following history of her case. She had been of a feeble constitution from infancy: three years ago her health became impaired, and she was affected with that erratic appetite called by Good *limosis pica;* to gratify which, she had eaten for a long time, and in large quantities, magnesia, chalk, and clay: this complaint was attended with a troublesome headach, and other marks of derangement of the functions of the digestive organs.

She had menstruated irregularly, and the discharge was deficient in quantity and abnormal in quality. About a year ago she became affected with sharp pains through the abdominal and uterine regions, accompanied with heavy bearing-down sensations, like parturient contractions of the uterus; swelling and exquisite tenderness of the abdomen, and retention of urine, requiring the repeated use of the catheter. After some weeks' suffering, she was attacked with trismatic tetanus: the trismus continued forty-eight hours, during which time the disease came on in paroxysms; a trait that it has constantly exhibited in every attack.

The resolution of the first trismatic spasm was attributed to the use of prussic acid.

About this time there was a discharge of a considerable quantity of fœtid, purulent-looking matter, *per anum et vaginam;* and, after much suffering from this bearing-down sensation, conjoined with a pricking, tearing, or lacerating feeling, an angulated piece of earthy matter, of a light colour, interspersed with white spots, and of a hard and compact texture, was discovered in the vagina, and removed.

The second attack of trismus was about a month subsequent to the first, and continued between three and four days, and left her spontaneously. This was also succeeded by a dejection of this earthy matter, which the attending physician had already pronounced to be the product of "gravel of the womb."

The third attack was in September, seven or eight months subsequent to the second: it continued, with intervals of relief from the spasms, about a month.

In the intervals of the attacks of trismatic tetanus, she was not free from any of the concomitant affections, only suffering them in a less degree than seemed necessary to induce the tetanic spasms.

When she came under my care, she had been labouring under alternations of the spasms, and intervals of comparative ease, for about a week, during which time the violence of the spasms had been moderated by the laudanum and bleeding, the latter always producing a resolution of the trismatic spasm, when carried to the production of incipient deliquium; but the relief was very transitory. I directed her to take a tea-spoonful of the Oleum Terebinthinæ; Ext. Cicuta five grains; and Calomel five grains, alternately, every hour.

Next day, no return of the tetanus; freedom from the cramp and pains in the uterine region: a number of copious dejections from the bowels. Used the catheter, and removed some of the earthy matter from the vagina. Intervals of the medicine lengthened.

Two days afterwards I saw the patient: no return of tetanus; ptyalism; catheter used, and more earthy matter extracted from the posterior and superior part of the vagina; the matter was always found occupying the space between the cervix uteri, and the recto-vaginal septum; the projecting part of the cervix uteri being carried forward toward the symphysis pubis, the os uteri was always impervious to the finger, nor could any fissure or opening be detected by the most careful manual examination, though the relative situation and extreme tenderness of the parts were unfavorable to a satisfactory investigation.

The patient continued exempt from any tetanic affection about ten days; the gastric and abdominal pains being rendered tolerable by the free use of Ext. Cicuta, which, when used in sufficient quantities to relieve the pain, was productive of derangement of the cerebral functions.

During this period, a hard circumscribed tumor in the right hypogastric region was discovered, occupying a line drawn from the umbilicus to the superior anterior spinous process of the ilium of the corresponding side, exquisitely tender, hard, and inelastic, which was asserted to have been noticed for a long period. She was now directed to keep the bowels open, and to use the turpentine and cicuta pro re nata.

It should be remarked, that every attack of tetanus, succeeded by a dejection of earthy matter, was followed by a copious secretion of milk, and a number of times by the formation of an abscess in the breast.

In a few weeks the patient was able to visit me, a distance of eight miles; but she suffered much abdominal pain, produced by the motion of the carriage.

The second day subsequent to her visit, I was desired to see her; when she informed me by writing, her jaws being locked, that her suffering from excruciating pain had been inexpressibly severe ever since her ride; that she felt a consciousness that her situation was more perilous, and the event more precarious, than it had ever yet been; and that this opinion arose from her feelings being so different from any she had ever before experienced; that the abdominal tumor had descended; that she suffered powerful, but ineffectual, expulsive pains, and a sensation as if a large body was tearing and forcing its way through a narrow aperture.

The spasm of the jaws was relieved by bleeding, and the same prophylactics used as before: but the latter were unavailing, notwithstanding sufficient quantities of calomel were introduced to excite a smart degree of ptyalism.

After bleeding was deemed improper, suppositories, containing about twenty grains of pulverised tobacco, with five grains of opium, were directed to be used per anum. The use of the first, in about two hours, was followed by nausea, vomiting, and a solution of the trismatic spasm of forty-eight hours standing. She had before objected to the exhibition of enemata, and she was now averse to the use of suppositories, from the local distress they occasioned: she was therefore desired to use the same remedies per vaginam, which she did a few times with success; but this form soon became ineffectual, but not till she had repeatedly been annoyed by the taste of tobacco in the mouth at every supervention of vomiting subsequent to the introduction of the remedy. A piece of tobacco was afterwards employed in the same way, with a ligature around it, by which it could be easily withdrawn on the accession of the vomiting, and with the same success as the preceding.

The last form also soon proved ineffective. It was now found that the remedies used for the tetanic affection, paralyzed those expulsive efforts by which the dejection of the earthy matter was effected: it was therefore deemed advisable to suffer the disease, as far as was compatible with safety, to take its own course, with the hope that the expulsive efforts would prove adequate to the dejection of the earthy matter, and thereby put a period to the paroxysms, as the descent of that substance was always the exciting cause of the tetanus.

By the use of the fore-mentioned means, the pains and spasms were a little mitigated for a few days, and twice the jaws were opened by the tobacco: once for about twenty minutes, in which time she drank a tumbler of water; another time for about fifteen minutes, during which she was unable to take any thing, by reason of the distressing nausea at the stomach, occasioned by the tobacco.

With the exception of these intervals, the jaws were uninterruptedly closed eight days and eight nights, the tetanic spasms harassing her, at more or less distant intervals, and the abdominal pains continuing excruciating.

During the first few days, the severity of suffering seemed slightly mitigated by the remedies used, but they all soon lost their effect. She had now lain eight days without food or drink, save one draught of water, and suffering the most exquisite torture, and the vital powers now appeared rapidly sinking. In this situation, it was determined to make one desperate effort to rescue the patient from this terrible disease, thinking with Celsus, that "satius est enim anceps auxilium experiri quam nullam;" and knowing the powers of tobacco in prostrating the powers of animal life, the conviction forced itself upon my mind, that, if I could subdue these powers, it would then be practicable to subdue the disease. It was therefore determined to make a cautious repetition of the exhibition of the tobacco, in small quantities, until some powerful effect was wrought either on the subject or the disease. An infusion of the article was prepared, and a part injected per anum, and a part per vaginam, and the latter retained by closing the orifice with a cloth.

This double application was repeated once an hour to the third time, when unequivocal evidence of its effects showed itself: the patient became pale and cold; tremor of the extremities; a small feeble pulse; anxiety and oppression at the præcordia, with efforts to vomit, soon followed by powerful vomiting, and a gradual opening of the jaws.

This seemed to break the charm; for although she had many slight returns of the trismus, yet not one exceeding a few hours in duration, and this without any tetanus.

Large and repeated dejections of the earthy matter* now took place in rapid succession, and she gradually recovered from every symptom of tetanus, and has continued exempt to the present time, a period of about five years. She has had no dejections of the earthy materials since convalescence from that attack. The tumor in the hypogastric region has also disappeared, with all those distressing pains in that region

* A specimen of this matter accompanies this communication. It bears a perfect resemblance, in all its external and sensible characters, to the clay found between the backs of old chimneys, even in the empyreumatic smell that the clay in such situations imparts, from its impregnation with smoke. This peculiar smell of the clay was very perceptible soon after it became dry. Some of the pieces of clay contained minute splinters of wood and straw intermixed. The patient asserted that she had been in the habit of often picking pieces of dried clay out from between the bricks of the chimney, and eating it. The aggregate quantity of this earth, which was evacuated per vaginam, is estimated to have measured about two quarts.

and in the uterus. Menstruation is more regular and better in quality, but still she is somewhat affected with the limosis pica, but she abstains from gratifying this appetite. Her general health is feeble, but she is free from the burden which so long incommoded her by its weight and size.

In this case, the purulent discharge per anum et vaginam, preceding the dejection of the earthy matter into the vagina, furnishes a clue to the manner in which this matter arrived there; and the previous habit of the patient in eating earths, and the abdominal tumor, render it probable that an accumulation of these earthy substances took place in some portion of the intestinal canal, forming a sac which eventually became so occluded from the intestinal canal, as to prevent the fæces passing this way, and its weight causing it to descend into the pelvis, where adhesion to the surrounding parts took place, and finally ulceration, by which a passage was formed, through which the earthy matter made its exit into the vagina.

A case of the formation of a sac by the coats of the intestines, and occlusion from the common canal, by taking a large quantity of an amalgam of quicksilver, is reported in some of the early numbers of the Philadelphia Journal of the Medical and Physical Sciences, which bears some resemblance to the manner in which I have supposed the earthy matter to have become occluded from the alimentary canal.

*Kennebunk, Maine; March* 1830.

